# An Evaluation of Experimental Calcium Ion-Leachable Nanocomposite Glass Ionomer Cements

**DOI:** 10.3390/nano13192690

**Published:** 2023-09-30

**Authors:** Ioannis Tsolianos, Alexandros K. Nikolaidis, Elisabeth A. Koulaouzidou, Dimitris S. Achilias

**Affiliations:** 1 Division of Dental Tissues’ Pathology and Therapeutics (Basic Dental Sciences, Endodontology and Operative Dentistry), School of Dentistry, Aristotle University Thessaloniki, 541 24 Thessaloniki, Greece; itsolianos@gmail.com (I.T.); koulaouz@dent.auth.gr (E.A.K.); 2 Laboratory of Polymer and Color Chemistry and Technology, Department of Chemistry, Aristotle University Thessaloniki, 541 24 Thessaloniki, Greece; axilias@chem.auth.gr

**Keywords:** glass ionomer cement, montmorillonite, Ca-clay nanoparticles, nanoclay, scanning electron microscopy, X-ray diffraction, acid–base reaction, compressive strength, calcium release

## Abstract

Glass ionomer cements (GICs) are among the main restorative dental materials used broadly in daily clinical practice. The incorporation of clay nanoparticles as reinforcing agents is one potential approach to improving GIC properties. This study aims to investigate whether the incorporation of calcium-modified clay (Ca-clay) nanoparticles in conventional GICs alters their structural characteristics, along with their physicochemical and mechanical properties. Scanning electron microscopy (SEM) and X-ray diffraction (XRD) analyses were performed to assess the surface characterization of GIC nanocomposites, whereas a setting reaction was carried out via an attenuated total reflection Fourier transform infrared spectrometer (ATR-FTIR). A universal testing machine was used for compression tests, while calcium ion release was quantified using inductively coupled plasma optical emission spectrometry (ICP-OES). GIC composite groups reinforced with Ca-clay were found to release a fine amount of calcium ions (5.06–9.91 ppm), with the setting reaction being unaffected for low Ca-clay loadings. The median compressive strength of 3 wt% in the Ca-clay group (68.97 MPa) was nearly doubled compared to that of the control group (33.65 MPa). The incorporation of Ca-clay nanoparticles in GICs offers a promising alternative among dental restorative materials regarding their chemical and mechanical properties.

## 1. Introduction

It is common knowledge that glass ionomer cements (GICs) are considered to be classic materials with regard to restorative dentistry. Conventional GICs were initially presented in 1972 by Wilson and Kent [1,2,3]. They are composed of three kinds of substances, which are water, water-soluble acid, and basic glass [4]. Their mixing procedure may involve either hand mixing with a spatula or capsule mixing [5]. After 2–3 min, an acid–base reaction takes place, where a polyacid solution is linked to glass cations [4]. As a restorative material, glass ionomer cements offer a good chemical adhesion, biocompatibility, release of fluoride, stability of dimensions, and coefficient of thermal expansion close to teeth [2,3,6,7]. Among their various clinical applications, they can be used for primary dentition restorations, cementations, or as liners [2,4].

Concerning the properties of interest, international standards (ISO 9917:1991, ISO 9917-1:2007) define in detail which properties are important in conventional GICs. These are compressive strength, (radi)opacity, optical properties, acid erosion, and solubility, as well as setting time, and have been evaluated by a large amount of studies so far [8,9,10,11,12]. However, despite their advantages, GICs possess a low mechanical strength, brittleness, sensitivity to moisture, low wear resistance, and fracture toughness [2,3,13]. To face these issues, two different strategies have been attempted, involving the incorporation of reinforcing agents (metal, fibers, and hydroxyapatite) and the inclusion of polymerizable dimethacrylate monomers that undergo photo-polymerization (resin-modified GICs) [14,15]. The addition of resin monomers alters the mechanism of reaction, where photo-polymerization occurs via light activation [2,4]. Resin-modified GICs are characterized not only by the maintenance of conventional GICs’ advantages, but also by their ease of handling, improvement in mechanical properties, and prevention against secondary caries [2,6,16], thus being an improved clinical alternative of conventional GICs [6].

Bioactivity seems to be a preferable property for restorative materials too. According to the British Standards Institution (BSI, London, UK), a material is called bioactive when it is able to induce apatite formation on the surface of a material, in close proximity with body fluids or living tissues [17,18]. In restorative dentistry, bioactive materials may induce hard tissue remineralization, the formation of apatite-like structures, or tissue regeneration [19]. The use of GICs, in general, is one of the main applications of bioactivity in restorative dentistry [20,21], as they are characterized by fluoride release, remineralization, and chemical adhesion [4,22]. Furthermore, bioactive glasses (BAGs) also possess bioactive properties, as their use enhances the formation of apatite in dentin, leading to dentin remineralization [23]. Calcium-silicate-based materials induce calcium release and the fast formation of apatite, acting as scaffolds towards dentin reparation, making them famous bioactive compounds too [24].

The application of nanotechnology to restorative dental materials could further reinforce not only their bioactivity [16], but also their mechanical, esthetic, and handling properties, as well as their wear resistance [25,26,27,28]. Clay minerals with a nanostructure such as montmorillonite (MMT) have become increasingly popular in dentistry. It is a layered phyllosilicate [29], 2:1 sheet structure, in which two layers (octahedral and tetrahedral) are fused [29,30,31]. The incorporation of clay nanoparticles in dental restorative materials may have a positive effect on their mechanical, thermal, and adhesive properties, as well as on ion exchange and their antibacterial activity [9,32,33]. Because of its unique structure, nanoclay is characterized by a big surface area, promoting adhesion and improved mechanical properties [29,31,34]. In order to provide better antibacterial properties, scientists have attempted to enrich MMT clay with calcium cations (Ca-clay), as the latter are considered to be agents for remineralization [32].

In terms of restorative materials, so far, Ca-clay has been introduced into composite resins and conventional GICs [32,35]. In conventional GICs, clay nanoparticles have also been applied, where Fareed and Stamboulis concluded that the mechanical properties were improved [9]. Based on the existing literature, it needs to be investigated whether the incorporation of Ca-clay into conventional GICs causes opposite effects in the different properties of these materials.

The aim of this study is to highlight whether the incorporation of Ca-clay nanoparticles in GICs might improve calcium release and have an effect on their chemical and mechanical performance. For this purpose, natural clay (Na-clay) nanoparticles were initially converted into their Ca-clay counterparts. The latter were inserted into a conventional GIC and the produced nanocomposites were examined in terms of their structural characteristics, acid–base reaction mechanism, calcium release ability, and compressive strength.

## 2. Materials and Methods

### 2.1. Materials

A commercially available conventional glass ionomer cement was selected as a restorative dental material of interest, namely GC Fuji^TM^ II radiopaque glass ionomer restorative cement (shade: No. 22-yellow brown, GC Corporation, Tokyo, Japan). Plate-shaped nanoparticles of raw hydrophilic bentonite clay (Na-clay), with the trade name Nanomer^®^ PGV (CEC value 145 × 10^−5^ eq/g), were produced by Nanocor Company and supplied by Aldrich. The chemical composition (in mass %) of the Na-clay was 62.9% SiO_2_, 19.6% Al_2_O_3_, 3.35% Fe_2_O_3_, 3.05% MgO, 1.68% CaO, and 1.53% Na_2_O, respectively [36]. Calcium chloride, CaCl_2_, (anhydrous, granular ≥ 93.0%) was also provided by Sigma-Aldrich, St. Louis, MO, USA. The calcium standard solution for ICP (1000 ± 2 mg/L) in 2% *w*/*w* HNO_3_ was used as a stock solution for the plotting of the Ca calibration curve, and was supplied by Sigma-Aldrich. HNO_3_, 65% *w*/*w*, (ultrapure) provided by Chem-Lab was diluted to 2% *w*/*w* with deionized water and used for Ca standard dilutions.

### 2.2. Clay Modification via Ion Exchange Reaction

The process was carried out by stirring 5 g of Na-clay in 250 mL of CaCl_2_ aqueous solution (0.5 M) for 24 h at 23 °C [37]. The product was then filtered using a G4 filter under vacuum and placed in a new solution. This process was repeated three times. After that, the material was washed three times with deionized water, dried in an oven for 24 h at 100 °C, and finally crushed into a powder (Ca-clay) using a mortar and pestle.

### 2.3. Preparation of Cement Pastes

Five different types of tested materials were prepared, based on their specific compositions (Table 1). The initial pastes were obtained according to the manufacturer’s instructions. The nanomaterial powder was weighed using an electronic weighing scale (Sartorius TE 1245) and embedded into the cement powder before mixing it with the cement liquid. The mixture was further agitated for 2 additional minutes until a uniform paste was gained.

### 2.4. Measurements

#### 2.4.1. Characterization of GIC Nanocomposites Morphology

Scanning electron microscopy (SEM) was carried out using a field emission scanning electron microscope, JEOL JSM-7610F Plus (JEOL Ltd. Company, Akishima, Tokyo, Japan), supported by an Oxford AZTEC ENERGY ADVANCED X-act energy dispersive X-ray spectroscopy (EDS) system (Oxford Instruments plc, Abingdon, Oxfordshire, UK), using the following parameters: a 15–20 kV accelerating voltage and an 8–11 mm working distance at a ×150, ×500 and ×2000 magnification. All the studied surfaces (clay nanoparticles and set cements) were coated with carbon black to avoid charging under the electron beam. An elemental analysis of the Na-clay and Ca-clay nano-powders was initially conducted. In addition, five bar-shaped specimens corresponding to the cement pastes of different material compositions were prepared by filling a Teflon mold with the mixed paste. The mold surfaces were overlaid with glass slides covered with a Mylar sheet, in order to avoid air entrapping and the adhesion of the final set material. The assembly was held together with spring clips and the cements were left to set for 24 h. The prepared specimens were then divided into two subgroups. The first subgroup was placed at 37 °C, whereas the second was stored in deionized water (5 mL) for 1 week at 37 °C. The specimens were then isolated, left to dry at room temperature, and finally subjected to an SEM analysis.

An X-ray diffraction (XRD) analysis of the set cements (24 h) in the form of films was performed over the 2θ range from 2° to 12°, at steps of 0.05° and a counting time of 5 s per step, using a Miniflex II XRD system from Rigaku Co. (Tokyo, Japan) with Cu Ka radiation (*λ* = 0.154 nm). The clay nano-powders were also scanned under the same conditions.

#### 2.4.2. Assessment of the GIC Nanocomposites’ Setting Reaction Progress

For the evaluation of the extent of the acid–base reaction, pastes from each of the five experimental groups were placed between two glass plates, forming a circle surface. The samples were then analyzed by the means of an attenuated total reflection Fourier transform infrared (ATR-FTIR) spectrometer (Cary 630, Agilent Technologies, Mulgrave, Victoria 3170, Australia) in the 2000–500 cm^−1^ scanning range. The resolution of the equipment was 4 cm^−1^ and the number of scans was 32. Spectra acquisitions from the set materials (*n* = 4) were performed after 15 min, 1 h, 24 h, and 1 week following the mixing. Reference spectra of the deionized water were also recorded. The progress of the setting reaction was calculated on the basis of the peak height ratio of the Ca/Al carboxylate salts formed (1600–1500 cm^−1^) [38,39,40] to the remaining unionized carboxylic acid groups (~1700 cm^−1^) at specific time intervals, by utilizing a peak deconvolution process due to the interference of water molecule absorbance (1637 cm^−1^).

#### 2.4.3. Compression Tests

Cylindrical specimens (4 × 6 mm) for the compressive tests were prepared (*n* = 10), by filling a Teflon mold with the mixed paste in accordance with ISO 9917-1 [41]. The mold surfaces were overlaid with glass slides covered with a Mylar sheet, in order to avoid air entrapping and the adhesion of the final set material. The assembly was held together with spring clips and the cements were left to set for 10 min. Afterwards, they were stored at 37 ± 1 °C for 24 h and subsequently placed in plastic vials containing 5 mL of deionized water at 37 ± 1 °C for 1 week. The test specimens (*n* = 10) were then removed and fractured on a universal testing machine (Testometric AX, M350-10 kN, Testometric Co., Ltd., Rochdale, UK) The diameter of the specimens was measured using a caliper at three different points, in order to calculate the average diameter of each of the specimens prior to testing. All the measurements were carried out at a cross-head speed of 1 mm/min until fracture occurred. The maximum load was recorded and the compressive strength (σ) in MPa was calculated using the standard formula:

σ = F/(4πD^2^)

where: F is the maximum load (N) and D is the specimen diameter (mm).

#### 2.4.4. Determination of Calcium Ion Release by Means of Inductively Coupled Plasma Optical Emission Spectrometry (ICP-OES)

After the storage of the compression specimens in water, the aqueous leachates were isolated, diluted with 20 mL of HNO_3_ (2% *w*/*w*), and immediately filtered prior to the elemental analysis. The measurements were conducted by the means of a radial viewing ICP-OES system (Perkin Elmer, Optima 2100 DV, Waltham, MA, USA). The optimum instrumental conditions were set as: a 0.8 L min^−1^ nebulizer argon flow rate, 1300 W of incident power, and a 1.5 mL min^−1^ sample flow rate. A characteristic spectral line of 317.933 nm was selected to determine the amount of Ca ions. Three readings were conducted during the sample running. Each sample was prepared and analyzed five times (*n* = 5), and an average value was taken to obtain accurate results. Deionized water diluted with 20 mL of HNO_3_ (2% *w*/*w*) was also measured as a blank solution for the baseline correction. The calibration curve was plotted as a regression line (y = 56,326x + 11,275, r^2^ = 0.9999) by diluting the stock Ca standard solution in the appropriate concentration ranges. The detection (LOD) and quantitation (LOQ) limits were calculated as 0.0025 ppm and 0.0076 ppm, respectively.

### 2.5. Statistical Analysis

The assumption of normal distribution was investigated for the variables using the Shapiro–Wilk test. The Kruskal–Wallis test was used to compare compressive strength and calcium release between the five groups correspondingly. Bonferroni corrections were made to adjust for multiple tests. The statistical analysis was performed using «IBM SPSS Statistics 28». The statistical significance level was set at *p*-value ≤ 0.05.

## 3. Results and Discussion

### 3.1. Verification of Ion Exchange Reaction in Clay Nanoparticles

**Figure 1:**
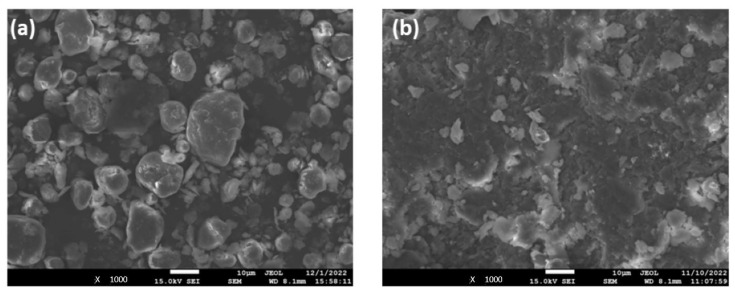
SEM images of: (**a**) Na-clay, and (**b**) Ca-clay nano-powders.

SEM images captured for the two examined types of clay nanoparticles (Na-clay and Ca-clay) can be seen in Figure 1. It is apparent that the morphology of the clay changed when the Na-clay was subjected to the ion exchange process. Specifically, more nanoclay clusters are observed in Figure 1a than in Figure 1b. The different electron configuration of Ca^2+^ ions increased the ionic size of Ca^2+^, thus amplifying the mutual cation repulsions between the clay intergalleries, and finally resulting in finer particles. The EDS analysis data (Table 2) reflect the median percentage values of the detected Ca^2+^ ions. It is clear that the Ca^2+^ levels found for the modified Ca-clay were approximately 3-fold higher compared to the Na-clay. This result may be an early indication that the ion exchange reaction within the interlamellar clay space extensively proceeded.

**Figure 2:**
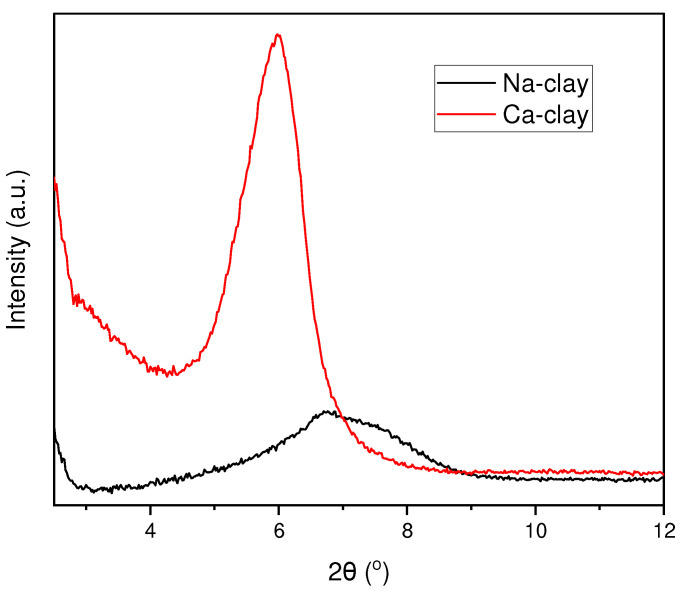
X-ray diffraction patterns of 2θ values between 2° and 10° highlighting the differences in d_001_-values between natural Na-clay and modified Ca-Clay nanoparticles.

X-ray diffractograms acquired for both the raw Na-clay and ion-exchanged Ca-clay nanoplatelets are presented in Figure 2. The recorded 2θ diffraction angles, as well as the respective d_001_ basal spacing values, calculated according to Bragg’s law (nλ = 2 dsinθ), are listed in Table 2. It is shown that the initial diffraction peak for the pristine clay (6.71°) was shifted to a lower angle (5.98°) after the insertion of Ca^2+^ ions within the intergallery space. It is obvious that the higher ionic size of Ca^2+^ in comparison to the Na^+^ cations provoked an interlamellar clay expansion corresponding to a d_001_ change of Δd_001_ = 0.16 nm. These results are in accordance with the SEM findings, confirming the successive ion exchange intercalation of the natural Na-clay.

### 3.2. Structural Characterization of GIC Nanocomposites

**Figure 3:**
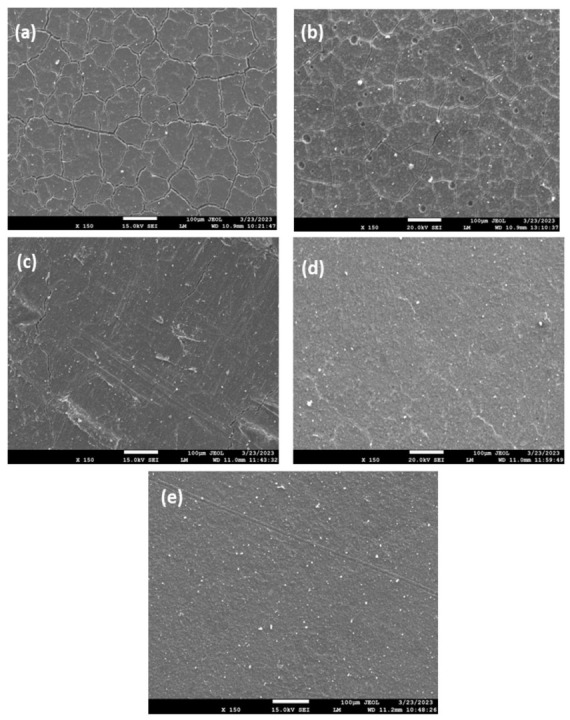
SEM images of (**a**) GIC (control), (**b**) GIC + 3 wt% Ca-clay, (**c**) GIC + 7.5 wt% Ca-clay, (**d**) GIC + 15 wt% Ca-clay, and (**e**) GIC + 15 wt% Na-clay.

**Scheme 1:**
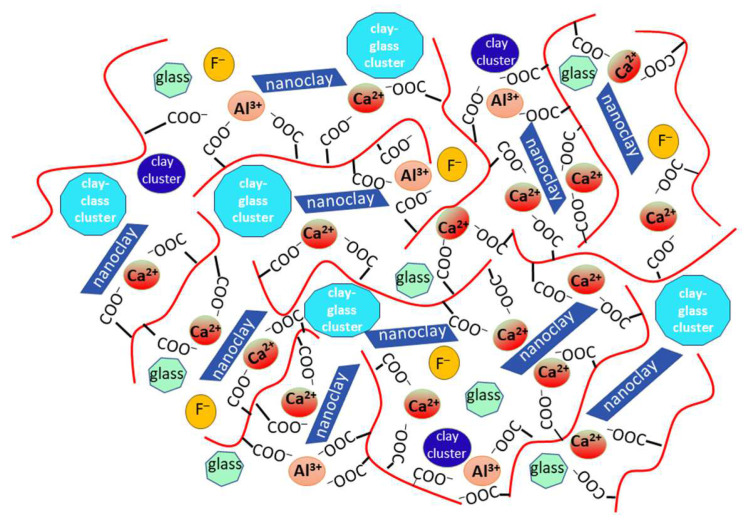
Schematic illustration of glass ionomer cement nanocomposite filled with Ca-clay nanoparticles (the red lines correspond to the macromolecular chains of poly(acrylic acid).

**Figure 4:**
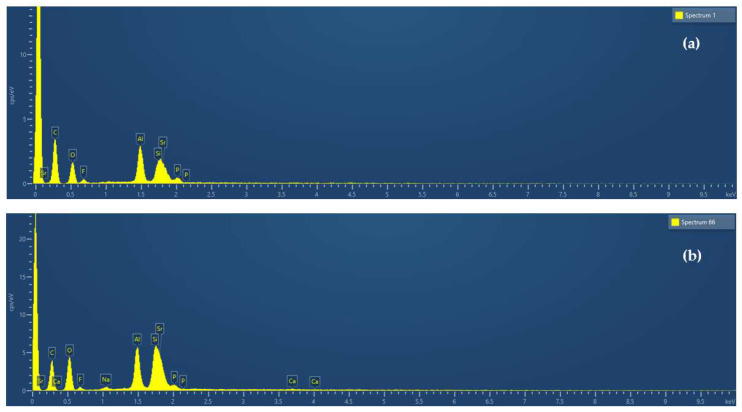

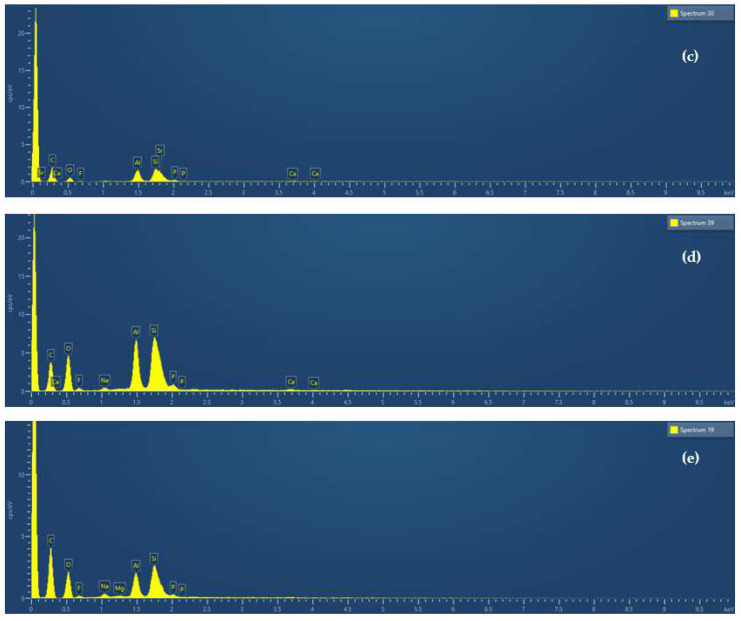
EDX spectra depicting elemental analysis of (**a**) GIC (control), (**b**) GIC + 3 wt% Ca-clay, (**c**) GIC + 7.5 wt% Ca-clay, (**d**) GIC + 15 wt% Ca-clay, and (**e**) GIC + 15 wt% Na-clay.

Figure 3 illustrates the SEM images of the control and nanocomposite GIC groups correspondingly. The dark regions are likely related to the poly(acrylic acid) matrix, while the localized white spots rather correspond to some inorganic clay and/or glass clusters distributed within the ionic crosslinked poly(acrylic acid) network of the GIC nanocomposites. An apparently adequate dispersion of filler clusters mainly represents the surface characteristics for the total of the studied dental materials. This structural feature may have derived from the hand-mixing process of liquid–powder according to the manufacturer’s instructions. Moreover, inherent surface cracks originated from the starting GIC (Figure 3a) are still obvious in the nanocomposites filled with relatively low clay loadings (Figure 3b,c). Concerning the GIC + 15 wt% Ca-clay nanocomposite (Figure 3d), it can be stated that, as the concentration of clay nanoparticles increased, these cracks seemed to be limited stepwise, due to possible a “healing effect” based on the diffusion of Ca^2+^ cations from the bulk to the surface. Consequently, more Ca^2+^ species were available to react with the carboxyl groups of the poly(acrylic acid), hence further enhancing the bridging of the ionic crosslinked network on the surface of the nanocomposite. This information is in agreement with the study of Kantowicz et al. [42], where the incorporation of TiO_2_ nanoparticles resulted in less cracks. In addition, a larger number of filler clusters is visible by elevating the amount of nanoclay (Figure 3d,e). The latter tendency could be attributed to the probable presence of clay–clay and/or clay–glass agglomerates (Scheme 1), as the packing density of the nanocomposite could be disrupted, especially at high clay filler loadings. Taking into account the EDS spectra recorded on the surface of the GIC nanocomposites (Figure 4), the determined percentage values of Ca are given in Table 3. The obtained results range between 0.11 and 0.65% among the Ca-clay groups, confirming the presence of Ca-clay in the GIC matrix. Nevertheless, the inconsistency between the added amount of Ca-clay and the measured ratio of Ca on the surface of the GIC nanocomposite could be due to the formation of the described aggregates, which could affect the uniform detection of Ca content and generate quantitative limitations.

**Figure 5:**
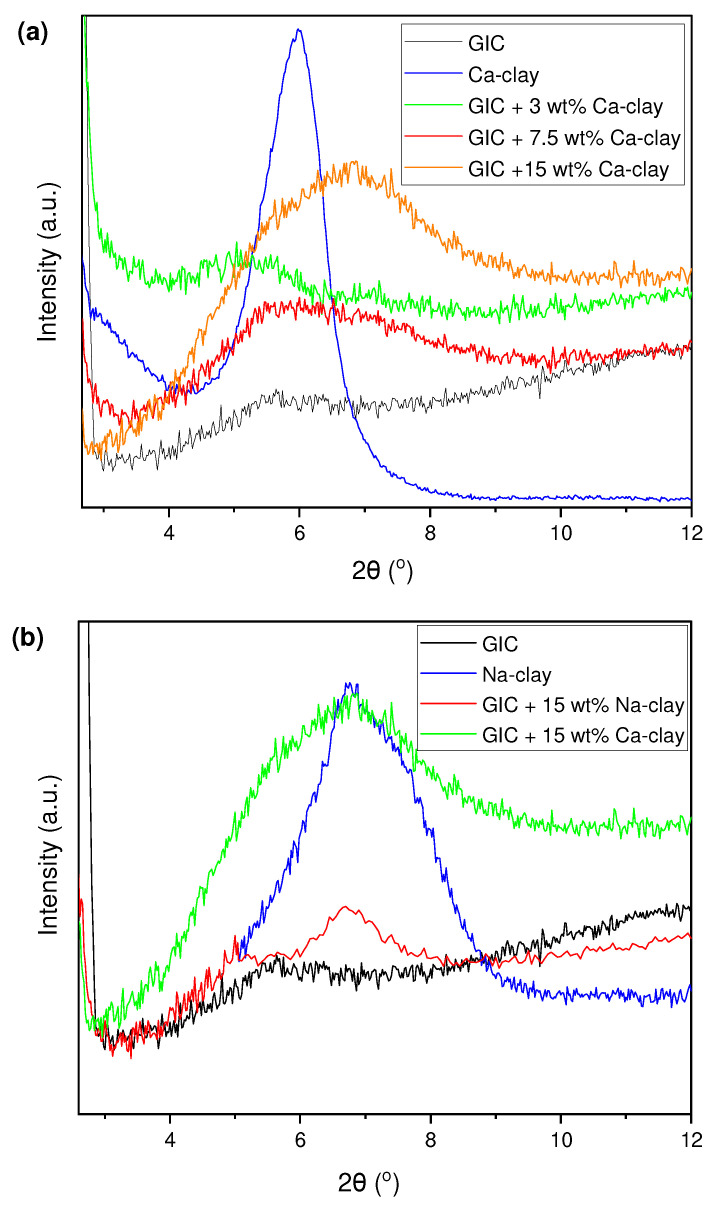
Comparative XRD spectra of GIC and GIC nanocomposites filled with (**a**) different amounts of Ca-clay nanoparticles, and (**b**) natural Na-clay and Ca-clay nanoparticles at the amount of 15 wt%.

Figure 5 demonstrates the XRD patterns for the Ca-clay and Na-clay nanofillers, as well as for their corresponding GIC nanocomposites and the conventional GIC. Moreover, the calculated interlamellar spacing d_001_ values of the nanoclay are accumulated in Table 3. It is clear that the addition of 3 wt% Ca-clay could shift the relative diffraction peak to a lower angle (Figure 5a), resulting in an increase in the d_001_ value (Δd_001_ = 0.27 nm) when comparing the data of Table 2 and Table 3. This tendency implies a possible intercalation of the poly(acrylic acid) macromolecular chains between the interlayer galleries of the Ca-clay. Dowling et al. reported that larger interlayer d_001_-spacings may facilitate the diffusion of poly(acrylic acid) chains within montmorillonite clay galleries [35]. In terms of the GIC + 7.5 wt% Ca-clay nanocomposite, the d_001_ augmentation was found to lower (Δd_001_ = 0.13 nm), as reflected by the occurrence of a diffraction peak between the Ca-clay powder and the nanocomposite containing 3 wt% Ca-clay (Figure 5a). Hence, a combination of intercalated clay layers along with some agglomerates could be justified at a 7.5 wt% nanofiller loading level (Scheme 1). However, a further reinforcement of the GIC with 15 wt% Ca-clay unexpectedly altered the pattern by shifting the diffraction peak to a higher 2θ angle region, namely almost at the same location with the Na-clay nanoparticles and GIC + 15 wt% Na-clay nanocomposite (Figure 5b). A possible explanation could be that, at such a high Ca-clay loading, the intensive swelling effect of the clay after being mixed with water may favor a probable migration of the inherent Ca^2+^ cations to the outer environment, so as to be available to interact with poly(acrylic acid), thus allowing for the smaller Na^+^ cations originated from the acid-attacked glass powder to enter the clay galleries. As a result, Na-clay tactoids could be formed inside the ionic crosslinked network, yielding a d_001_ value similar to that of the Na-nanoclay and its corresponding GIC nanocomposite (Table 2 and Table 3). The latter findings are in accordance with the SEM observations in terms of the structural features of the GIC nanocomposites revealed at high clay concentrations.

### 3.3. Evaluation of the Acid–Base Reaction

**Figure 6:**
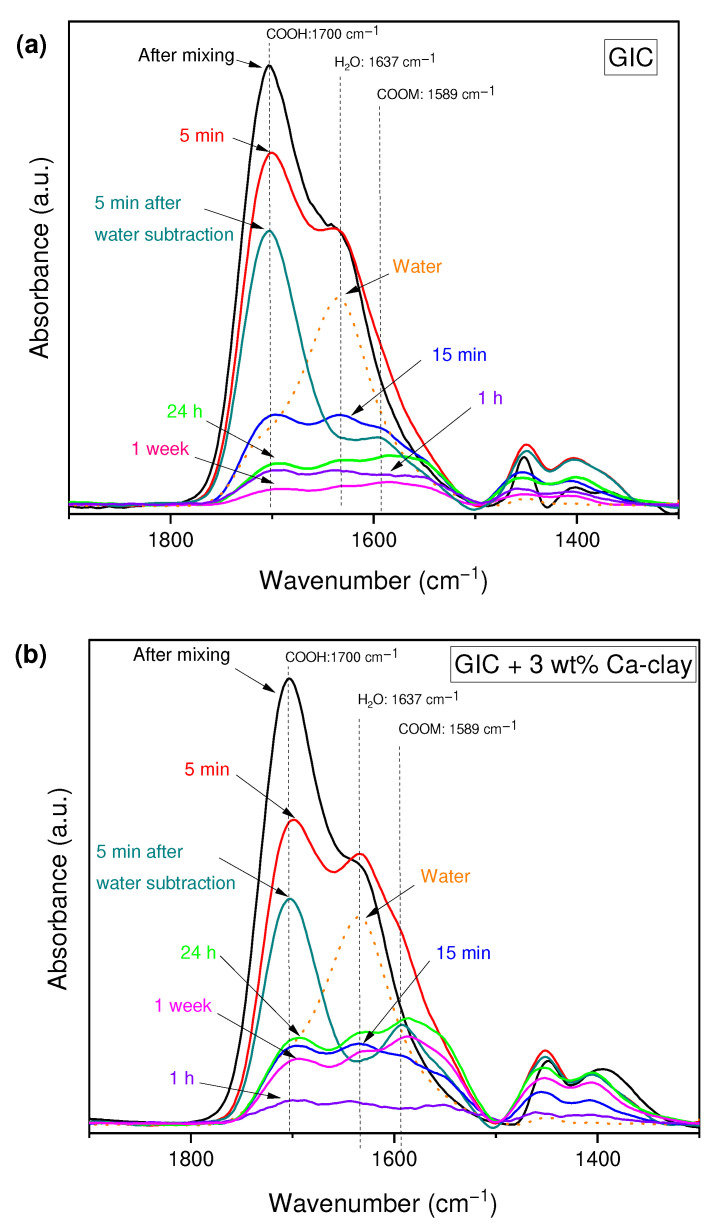

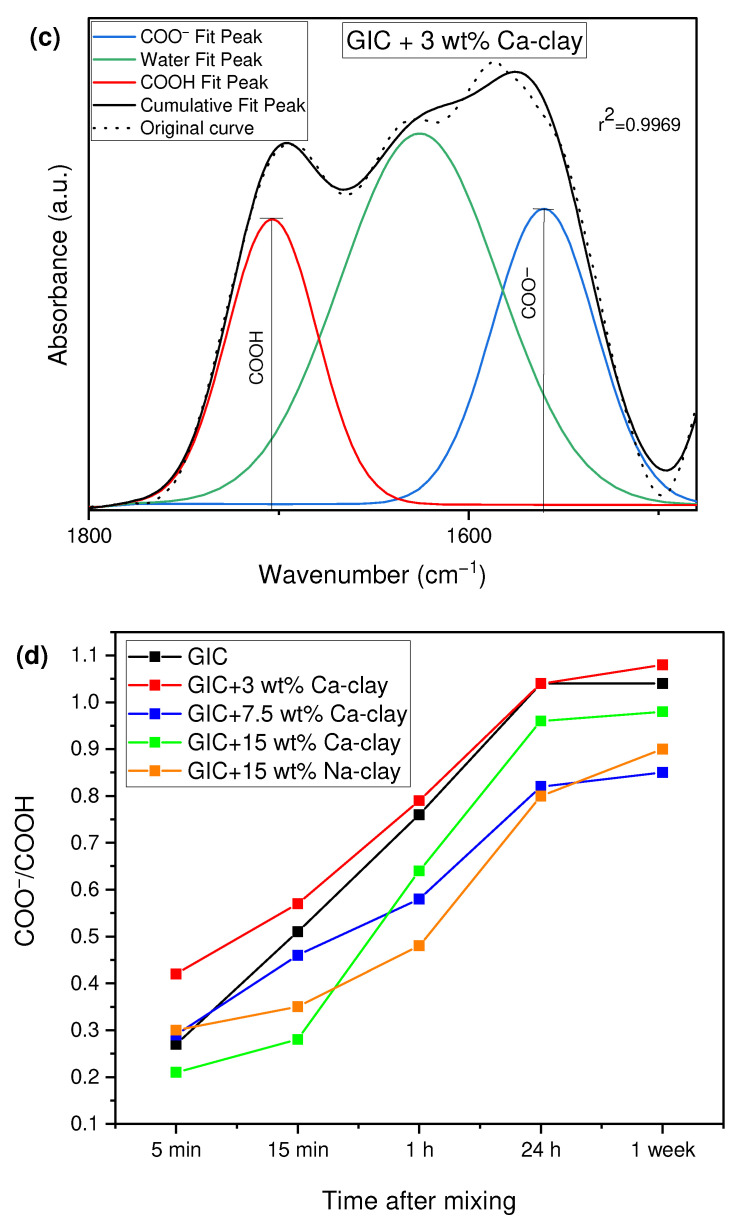
Representative ATR-FTIR spectra of: (**a**) Fuji II GIC (control) and (**b**) GIC + 3 wt% Ca-clay nanocomposite recorded at different time periods after powder–liquid mixing, indicating the presence of COOH and COO^−^ absorbance peaks, after digital subtraction of water spectrum, (**c**) original and Gaussian curve-fitted ATR-FTIR spectrum of GIC + 3 wt% Ca-clay nanocomposite, and (**d**) plots of COO^−^/COOH peak height ratio vs. time reflecting the extend of acid–base reaction for GIC nanocomposites loaded with different amounts of clay nanoparticles.

Figure 6a,b illustrate the typical ATR-FTIR spectra acquired at different time periods after the liquid–powder mixing for the control GIC and GIC + 3 wt% Ca-clay nanocomposite, respectively. The deconvolution method used for the calculation of the COO^−^/COOH ratio in all the recorded spectra is also presented for the GIC + 3 wt% Ca-clay nanocomposite (Figure 6c). According to the Table 1 information, the studied GICs consisted of a polyacrylic acid reactant for the polycarboxylate salt production, low-molecular-weight polybasic carboxylic acid acting as a setting controller agent [40], water for acid ionization, and Ca-Al-F ion-leachable glass particles. The setting reaction process is considered to be governed by diffusion-controlled phenomena into the aqueous environment and proceeds via two steps. The initial stage is characterized by an acidic attack of the polyacid protons to the basic sites of the glass particles, leading to the extraction of Ca^2+^, Na^+^, and finally Al^3+^ ions. Then, the polyacid carboxylic groups can react with the released ions, resulting in a rigid network due to the presence of ionic crosslinks associated with the formation of insoluble carboxylate salts. The physical properties of the produced GIC are improved through the subsequent slow step of maturation, where additional reactions occur and more water molecules become strongly attached to the cement structure [4]. In each case, the spectrum resulting from the subtraction of the water spectrum revealed the appearance of new absorption bands in the region of 1600–1500 cm^−1^ due to the formation of carboxylates even 5 min after mixing, thus implying the development of an acid–base reaction (Figure 6a,b). It is also observed that the peak intensity of the unionized carboxylic groups at (~1700 cm^−1^) reduced over time in relation to the absorbance of the formed carboxylate salts. The acid–base reaction kinetics of the studied Fuji II and GIC nanocomposites are presented in Figure 6d. Furthermore, the COO^−^/COOH ratio values determined at specific time intervals for the estimation of the extent of the acid–base glass ionomer reaction are listed in Table 4. The obtained data confirmed the expected accomplishment of the setting reaction within 24 h, as the measured ratios did not change even after 1 week for the total of the tested GIC nanocomposites. After 5 min of mixing, it was revealed that the GIC reinforcement with 3 wt% Ca-clay accelerated the reaction in the initial stages. Ca^2+^ ions are well-known to dominate the acid–base reaction, as they react faster than Al^3+^ during the first steps of GIC setting [39]. Provided that the presence of 3 wt% Ca-clay nanoparticles ensured a higher Ca/Si ratio than the pristine GIC, the Ca^2+^ abundance might have facilitated the reaction progress, leading to the maximum salt formation in the early stages. After 1 h of reaction, the majority of the Ca^2+^ species in the nanocomposite were consumed and the acid–base reaction was controlled by the less reactant Al^3+^ ions. Therefore, the ultimate yields for the GIC + 3 wt% Ca-clay nanocomposite were very close to the corresponding COO^−^/COOH values of the control GIC (Figure 6d). The aforementioned trend shows that the incorporation of Ca-nanoclay into a conventional GIC at a low filler loading does not affect the setting reaction of the GIC. As the clay concentration was elevated, it was clear that the reaction rate was delayed 15 min after the initial mixing of the liquid–powder components (Table 4). During this time period, the high levels of clay nanoplatelets could be responsible for the absorbance of the available water molecules supplied by the liquid component. Accordingly, the swelling effect of the nanoclay decreased the content of water required for the solubilization and ionization of the pulverized polyacrylic acid. These phenomena possibly limited the acid attack on the ion-leachable glass particles, and eventually affected not only the intermediate, but also the ultimate yields of the carboxylate salt formation in the nanocomposites’ structures. In addition, the presence of Ca^2+^-modified clay at 15 wt% could sustain the hardening process 1 h after mixing in comparison to natural clay, maybe due to the availability of Ca^2+^ capable of being involved in the setting reaction mechanism.

### 3.4. Evaluation of Mechanical Performance

**Figure 7:**
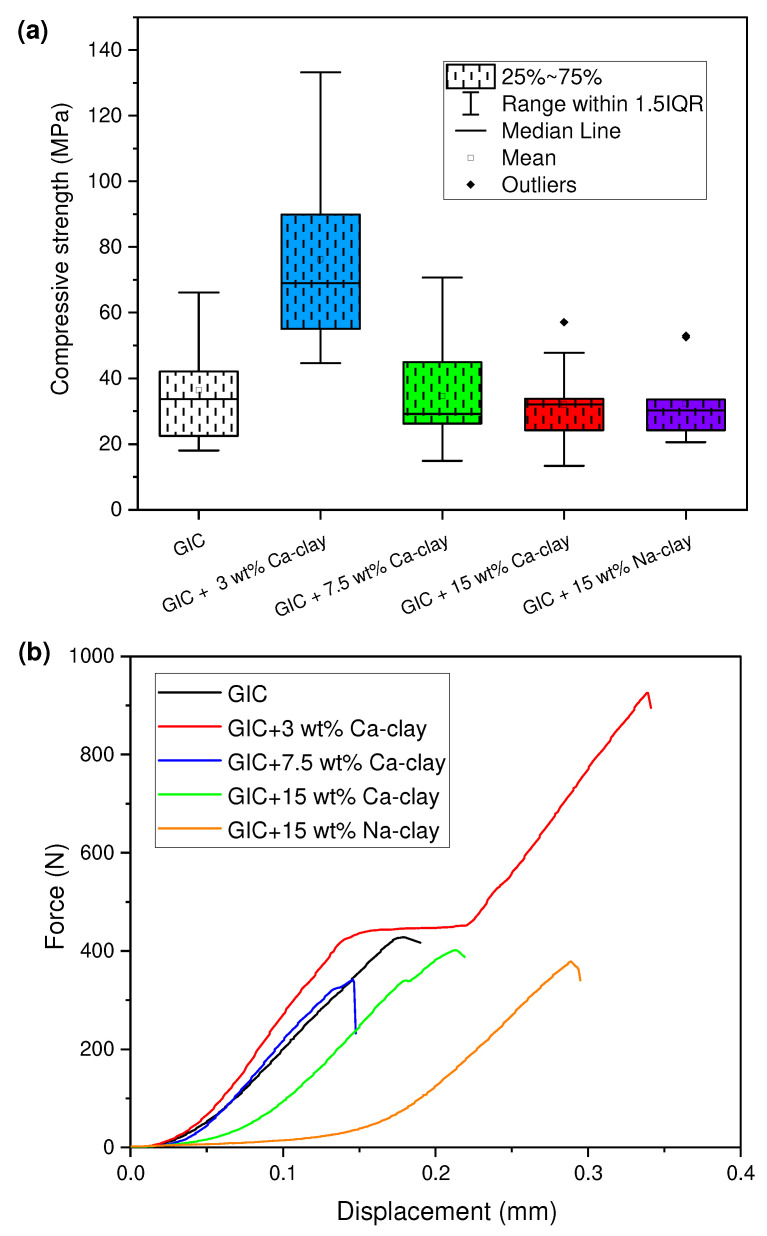
(**a**) Box plots of compressive strength and (**b**) graphic representation of force against the displacement, illustrating the effect of the nanoclay on the compressive resistance of GIC nanocomposites after 1 week storage in deionized water.

The strength values reflecting the mechanical resistance of the synthesized GIC nanocomposites against compressive stresses are presented in Table 5. Furthermore, Figure 7 presents the overall mechanical response of the tested nanocomposites. The obtained results ranged from 29.19 to 68.97 MPa. Gjorgievska et al. prepared GICs filled withAl_2_O_3_, ZrO_2_, and TiO_2_ nanoparticles, reaching compressive strengths within 32.34–47.35 MPa, under storage conditions similar to the present study [43]. Duarte et al. modified GICs with calcium phosphate nanoparticles, resulting in 21.80–39.20 MPa after 1 day of storage in water [44]. Higher strength values have been also reported by other experimental studies [10,45]. Undoubtedly, there are different mixing approaches to GICs based on studies in favor of either hand or capsule mixing but involving contradictory results. For instance, Nomoto and McCabe concluded that hand mixing is superior to capsule mixing due to the possible existence of air pores in capsule-mixed cement, which may reduce its compressive strength [5]. On the other hand, Oliveira et al. [46] measured a lower strength regarding hand-mixed conventional GICs. The above claims may account for the diversity in the literature data related to the levels of determined compressive strength. According to the Table 5 data, there were statistically significant differences in the compressive strength between the five groups (global *p*-value < 0.001). More specifically, the GIC + 3 wt% Ca-clay group had a significantly higher compressive strength than the GIC group (*p*-value = 0.013), the GIC + 15 wt% Ca-clay group (*p*-value = 0.003), the GIC + 15 wt% Ca-clay group (*p*-value = 0.005), and the GIC + 7.5 wt% Ca-clay group (*p*-value = 0.010). There were no significant differences between the rest of the groups.

It is worth pointing out that the incorporation of 3 wt% Ca-clay nanoparticles almost doubled the compressive strength of the conventional GIC (Figure 7a), with the simultaneous support of ultimate failure at high deformations (Figure 7b). It was previously stated that the extent of the acid–base reaction was not affected by the presence of low amounts of Ca-nanoclay, while intercalated clay conformations were confirmed by the XRD data as well. Under this regime, the intercalation of poly(acrylic acid) chains between the clay platelets could have contributed to the controlled transfer of the internal mechanical stresses from the macromolecular chains to the rigid nanoplateles, leading to the uniform distribution of exerted compressive forces throughout the ionic crosslinked network. Consequently, the crack propagation was delayed and the final displacement of GIC + 3 wt% Ca-clay nanocomposite increased in comparison to that of the other tested GICs. Moreover, Fareed and Stamboulis [47] found a little increase in the compressive strength of GICs at low nanoclay concentrations (1 wt%, 2 wt%) after 1 week of aging in distilled water at 37 °C, whereas for 4 wt% nanoclay, the handling process became uncomfortable and the compressive strength decreased. A similar attitude was observed in the current work for both the handling properties and ultimate strength, when the clay amount was over 3 wt%. When 15 wt% clay was added, it was found that the modification of clay with Ca^2+^ could sustain the compressive strength at slightly higher values relatively to the GIC reinforced with natural clay (Table 5), possibly due to the efficiency of the acid–base reaction (Table 4). In addition, an effort was made to insert up to 30 wt% Ca-clay, producing a quite firm paste, whereas the corresponding specimens did not withstand under the selected aging conditions and were eventually excluded from the studied groups.

### 3.5. Assessment of Calcium Ion Release

**Figure 8:**
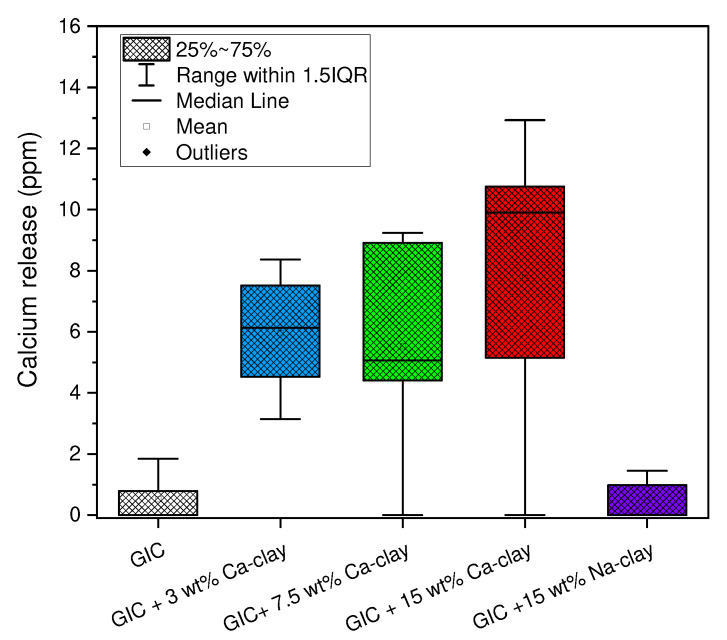
Comparative box plots representing the variation of calcium release for GIC nanocomposites after 1 week storage in deionized water.

Regarding the calcium release data acquired using the ICP-OES technique, even though there were differences between the five groups (global *p*-value = 0.015), the multiple two-by-two comparisons between the groups showed no significant differences (Table 6, Figure 8).

As is shown in Table 6, there were noticeable amounts of calcium ions detected in the aqueous extracts among the Ca-clay groups. Nicholson et al. determined slight amounts of calcium ions when commercial GICs were stored in deionized water in comparison to under acidic conditions [48]. These results are in agreement with the data in Table 6, where the calcium levels for the control GIC and the GIC filled with 15 wt% Na-clay were found to be negligible. Previous research work has also reported that the incorporation of TiO_2_, Al_2_O_3_, and ZrO_2_ nanoparticles (2–10 wt%) into GICs increased the Ca^2+^ ion release in physiological saline after 7 days [43]. However, the calcium concentrations ranged between 0.84 and 1.75 ppm, namely much lower than the calcium abundance determined in the present study. Herein, as the GICs were enriched with Ca-clay nanoparticles, a possible migration of highly available Ca^2+^ ions from the ionic crosslinked network to the outer aqueous environment might have favored the occurrence of calcium species at the level of approximately 5–10 ppm. Particularly, the presence of 3 wt% Ca-clay could lead to the release of 6.13 ppm Ca^2+^. At this clay loading, the surface cracks captured by SEM (Figure 3b) could support the diffusion phenomena associated with calcium release. Regarding the GIC + 7.5 wt% Ca-clay nanocomposite, possible structural conformations governed by surface crack healing (Figure 3c) or particle agglomerates (Scheme 1) could act as obstacles against the movement of calcium ions from the GIC bulk to the aqueous medium, thus limiting the rate of leaching to 5.03 ppm. Furthermore, when the relatively large quantity of 15 wt% Ca-clay was used, the enhanced pool of Ca^2+^ ions might have acted as a driving force toward their spatial movement, thus overcoming the above constraints and increasing the calcium ion release again. The aforementioned tendencies reveal that there was not an absolute correlation between the Ca-clay loading and the concentration of the released Ca^2+^, and it could be associated with the respective inconsistency that was previously confirmed by the SEM-EDS results acquired prior to the immersion of the GICs in the storage medium (Table 3).

On the other hand, Table 7 encompasses the SEM-EDS data related to the residual calcium identities accumulated on the surfaces of the GIC nanocomposites after their removal from the deionized water. It can be seen that the determined calcium percentage ranged within 0.37–1.48% in regard to the Ca-clay nanocomposite groups. Taking into account the corresponding values before the storage in water (Table 3), it is obvious that the calcium surface content decreased for the 3 wt% Ca-clay nanocomposite, while it increased for the GICs filled with 7.5 and 15 wt% Ca-clay, finally reaching a plateau of 1.45–1.48%. A possible explanation could be that, at high clay loadings, the provided abundance of Ca^2+^ ions may have favored the deposition of insoluble hydroxyapatite on the surface of the GIC, thus acting as a shield against further Ca^2+^ ion diffusion from the bulk to the external aqueous solution. Awosanya et al. proved that conventional, hand-mixed glass ionomer cements can release phosphate species in deionized water, namely orthophosphate PO_4_^3−^ and mono-fluorophosphate PO_3_F^2−^ ions, mainly due to dissolution rather than diffusion phenomena [49]. Furthermore, the silanol and carboxyl groups existing on the surfaces of GICs are considered to attract calcium and phosphorus ions, resulting in the nucleation of hydroxyapatite [50]. Consequently, the potential formation of a hydroxyapatite layer constitutes a different aspect related to the control of the calcium release, which could also justify the observed fluctuations in the concentration of the leached calcium by elevating the loading of the Ca-clay nanofiller.

### 3.6. Limitations of the Study

With regard to the limitations of this study, the GICs were hand mixed, although the SEM evaluation showed a homogenous distribution of the nano-powder on the GIC matrices. Hand mixing is also more clinically relevant, as dentists mainly use this mixing method in their daily clinical routine. As stated in terms of the compressive strength, the 30 wt% Ca-clay group was omitted, as the specimens were decomposed under the aging conditions. The handling of this nano-composite paste was more than difficult, while the transfer of this paste into the molds was also challenging.

In this in vitro study, deionized water was selected for the aging process of the specimens. However, a possible change of the liquid of interest could reinforce the clinical significance during an assessment of nanocomposite GIC surface modification and bioactivity. This could be attempted using Simulated Body Fluid (SBF) or Hank Balanced Salt Solution (HBSS) instead of deionized water, providing simulated clinical conditions. According to the existing literature, SBF or HBSS could be used for 28 days of aging in order to analyze apatite nucleation and surface modification. Analyses could be performed using SEM or XRD (as in the current study), before and after aging under simulated clinical conditions for 28 days. The aforementioned protocol has been used by various studies on endodontic sealers and polymeric scaffolds for bone regeneration, with reliable results [51,52,53].

## 4. Conclusions

In the present study, calcium-modified clay nanoparticles (Ca-clays) were successfully synthesized and subsequently inserted into commercially available glass ionomer cements (GICs). The SEM results revealed the presence of filler clusters, while, by increasing the nanoclay content, the observed surface cracks of the GIC nanocomposites were gradually reduced. According to the obtained XRD data, the lowest Ca-clay concentration (3 wt%) yielded intercalation structures, followed by the co-existence of inorganic aggregates when the amount of clay was further increased. At such a Ca-clay loading, the setting reaction was accelerated 5 min after mixing, but the final COO^−^/COOH ratio was found to be similar to that of the pristine GIC. Furthermore, the above composite exhibited a statistically significant improvement in compressive strength, whereas higher clay loadings did not alter the GICs’ mechanical performance. All the GIC nanocomposites containing Ca-clay nanoparticles presented a noticeable release of calcium ions after 1 week of storage in deionized water. These findings could promote future studies dedicated not only to the investigation of resin-modified GICs reinforced with Ca-clays at low nanofiller levels, but also to the investigation of these properties under simulated clinical conditions.

## Figures and Tables

**Table 1:** Description of the studied GIC nanocomposite formulations on the basis of their diverse composition.

Νο.	GIC Composition *			Abbreviated Name
1	Fuji II GC	Liquid	Poly(acrylic acid) Polybasic carboxylic acid: 10–20% Water: 50%	GIC
		Powder	Glass Oxide Poly(acrylic acid)	
2	Fuji II GC + 3 wt% Ca-clay			GIC + 3 wt% Ca-clay
3	Fuji II GC + 7.5 wt% Ca-clay			GIC + 7.5 wt% Ca-clay
4	Fuji II GC + 15 wt% Ca-clay			GIC + 15 wt% Ca-clay
5	Fuji II GC + 15 wt% Na-clay			GIC + 15 wt% Na-clay

* According to manufacturer’s sds data.

**Table 2:** Calcium percentage (*n* = 10), diffraction angles, and d_001_-values measured with EDS and XRD, respectively, on natural and calcium-modified nanoclay.

Nano-Powder	Median (IQR) (%)	2θ (deg)	d_001_ (nm)
Na-clay	0.73 (0.43)	6.71	1.32
Ca-clay	2.05 (0.63)	5.98	1.48

**Table 3:** Calcium percentage (%) on glass ionomer cement nanocomposites measured with EDS analyzer (*n* = 4), and d_001_-spacing values of the dispersed Ca-clay nanoparticles calculated on the basis of X-ray diffraction (XRD) spectra.

GIC Nanocomposite	Ca (%) Median (IQR)	d_001_ (nm)
GIC	-	-
GIC + 3 wt% Ca-clay	0.65 (1.09)	1.75
GIC + 7.5 wt% Ca-clay	0.11 (0.76)	1.61
GIC + 15 wt% Ca-clay	0.54 (0.87)	1.30
GIC + 15 wt% Na-clay	-	1.32

**Table 4:** Mean values (standard deviation) of the COO^−^/COOH ratio determined for the tested GIC nanocomposites.

GIC Nanocomposite	Time after Powder-Liquid Mixing				
	5 min	15 min	1 h	24 h	1 week
GIC	0.27 (0.05)	0.51 (0.10)	0.76 (0.22)	1.04 (0.03)	1.04 (0.08)
GIC + 3 wt% Ca-clay	0.42 (0.08)	0.57 (0.11)	0.79 (0.21)	1.04 (0.10)	1.08 (0.12)
GIC + 7.5 wt% Ca-clay	0.29 (0.16)	0.46 (0.23)	0.58 (0.18)	0.82 (0.04)	0.85 (0.05)
GIC + 15 wt% Ca-clay	0.21 (0.09)	0.28 (0.15)	0.64 (0.24)	0.96 (0.14)	0.98 (0.13)
GIC + 15 wt% Na-clay	0.30 (0.10)	0.35 (0.13)	0.48 (0.15)	0.80 (0.16)	0.90 (0.18)

**Table 5:** Median values (interquartile range, IQR) of compressive strength between the five groups.

GIC Nanocomposite	Compressive Strength (MPa) Median (IQR)
GIC	33.65 (22.48, 42.08) ^a^
GIC + 3 wt% Ca-clay	68.97 (55.08, 89.88) ^b^
GIC + 7.5 wt% Ca-clay	29.19 (26.25, 44.89) ^a^
GIC + 15 wt% Ca-clay	32.09 (24.25, 33.81) ^a^
GIC + 15 wt% Na-clay	30.26 (24.29, 33.62) ^a^

Different superscript indicates statistically significant difference (*p* < 0.05).

**Table 6:** Median values (ppm) of calcium release between the five groups.

GIC Nanocomposite	Calcium Release (ppm) Median (Range)
GIC	0 (0, 1.85) ^a^
GIC + 3 wt% Ca-clay	6.13 (3.15, 8.37) ^a^
GIC + 7.5 wt% Ca-clay	5.06 (0, 9.24) ^a^
GIC + 15 wt% Ca-clay	9.91 (0, 12.93) ^a^
GIC + 15 wt% Na-clay	0 (0, 1.45) ^a^

Different superscript indicates statistically significant difference (*p* < 0.05).

**Table 7:** Calcium percentage (%) on glass ionomer cement nanocomposites after 7 days of aging in deionized water, measured with EDS analyzer (*n* = 4).

Glass Ionomer Cement Nanocomposite	Median (IQR) (%)
GIC	-
GIC + 3 wt% Ca-clay	0.37 (0.18)
GIC + 7.5 wt% Ca-clay	1.45 (0.7)
GIC + 15 wt% Ca-clay	1.48 (0.52)
GIC + 15 wt% Na-clay	-

## Data Availability

Data available upon request.

## References

[1] Wilson A.D., Kent B.E. (1971). The Glass-Ionomer Cement, a New Translucent Dental Filling Material. J. Appl. Chem. Biotechnol..

[2] Almuhaiza M. (2016). Glass-Ionomer Cements in Restorative Dentistry: A Critical Appraisal. J. Contemp. Dent. Pract..

[3] Nagaraja Upadhya P., Kishore G. (2005). Glass Ionomer Cement—The Different Generations. Trends Biomater. Artif. Organs.

[4] Sidhu S.K., Nicholson J.W. (2016). A Review of Glass-Ionomer Cements for Clinical Dentistry. J. Funct. Biomater..

[5] Nomoto R., McCabe J.F. (2001). Effect of Mixing Methods on the Compressive Strength of Glass Ionomer Cements. J. Dent..

[6] Croll T.P., Bar-Zion Y., Segura A., Donly K.J. (2001). Clinical Performance of Resin-Modified Glass Ionomer Cement Restorations in Primary Teeth: A Retrospective Evaluation. J. Am. Dent. Assoc..

[7] Xie D., Brantley W.A., Culbertson B.M., Wang G. (2000). Mechanical Properties and Microstructures of Glass-Ionomer Cements. Dent. Mater..

[8] Brzović Rajić V., Ivanišević Malčić A., Bilge Kütük Z., Gurgan S., Jukić Krmek S., Miletic I. (2019). Compressive Strength of New Glass Ionomer Cement Technology Based Restorative Materials after Thermocycling and Cyclic Loading. Acta Stomatol. Croat..

[9] Fareed M.A., Stamboulis A. (2014). Effect of Nanoclay Dispersion on the Properties of a Commercial Glass Ionomer Cement. Int. J. Biomater..

[10] Howard L., Weng Y., Xie D. (2014). Preparation and Evaluation of a Novel Star-Shaped Polyacid-Constructed Dental Glass-Ionomer System. Dent. Mater..

[11] Mallmann A., Oliveira Ataíde J.C., Amoeda R., Rocha P.V., Jacques L.B. (2007). Compressive Strength of Glass Ionomer Cements Using Different Specimen Dimensions. Braz. Oral Res..

[12] Tsuzuki F.M., Pascotto R.C., Malacarne L.C., Bento A.C., Medina Neto A., de Castro-Hoshino L.V., Souza M., Nicholson J.W., Baesso M.L. (2021). Studies of the Early Stages of the Dynamic Setting Process of Chemically Activated Restorative Glass-Ionomer Cements. Biomater. Investig. Dent..

[13] Sidhu S.K., Watson T.F. (1995). Resin-Modified Glass Ionomer Materials. A Status Report for the American Journal of Dentistry. Am. J. Dent..

[14] Ching H.S., Luddin N., Kannan T.P., Ab Rahman I., Abdul Ghani N.R.N. (2018). Modification of Glass Ionomer Cements on Their Physical-Mechanical and Antimicrobial Properties. J. Esthet. Restor. Dent..

[15] Fierascu R.C. (2022). Incorporation of Nanomaterials in Glass Ionomer Cements-Recent Developments and Future Perspectives: A Narrative Review. Nanomaterials.

[16] Özcan M., Garcia L.d.F.R., Volpato C.A.M. (2021). Bioactive Materials for Direct and Indirect Restorations: Concepts and Applications. Front. Dent. Med..

[17] PAS (Publicly Available Specification) 132 (2007). Terminology for the Bio-Nano Interface.

[18] Gandolfi M.G., Taddei P., Siboni F., Modena E., Ciapetti G., Prati C. (2011). Development of the Foremost Light-Curable Calcium-Silicate MTA Cement as Root-End in Oral Surgery. Chemical-Physical Properties, Bioactivity and Biological Behavior. Dent. Mater..

[19] Hamdy T.M. (2018). Bioactivity: A New Buzz in Dental Materials. EC Dent. Sci..

[20] Ladino L.G., Bernal A., Calderón D., Cortés D. (2021). Bioactive Materials in Restorative Dentistry: A Literature Review. SVOA Dent..

[21] Skallevold H.E., Rokaya D., Khurshid Z., Zafar M.S. (2019). Bioactive Glass Applications in Dentistry. Int. J. Mol. Sci..

[22] Spagnuolo G. (2022). Bioactive Dental Materials: The Current Status. Materials.

[23] Fernando D., Attik N., Pradelle-Plasse N., Jackson P., Grosgogeat B., Colon P. (2017). Bioactive Glass for Dentin Remineralization: A Systematic Review. Mater. Sci. Eng. C.

[24] Gandolfi M.G., Siboni F., Botero T., Bossù M., Riccitiello F., Prati C. (2015). Calcium Silicate and Calcium Hydroxide Materials for Pulp Capping: Biointeractivity, Porosity, Solubility and Bioactivity of Current Formulations. J. Appl. Biomater. Funct. Mater..

[25] Barot T., Rawtani D., Kulkarni P. (2021). Nanotechnology-Based Materials as Emerging Trends for Dental Applications. Rev. Adv. Mater. Sci..

[26] Sasalawad S.S., Naik S.N., Shashibhushan K.K., Poornima P. (2014). Nanodentistry: The next Big Thing Is Small. Int. J. Contemp. Dent. Med. Rev..

[27] Sokołowski K., Szynkowska M.I., Pawlaczyk A., Łukomska-Szymańska M., Sokołowski J. (2014). The Impact of Nanosilver Addition on Element Ions Release Form Light-Cured Dental Composite and Compomer into 0.9% NaCl. Acta Biochim. Pol..

[28] Mantri S.S., Mantri S.P. (2013). The Nano Era in Dentistry. J. Nat. Sci. Biol. Med..

[29] Katti K.S., Jasuja H., Jaswandkar S.V., Mohanty S., Katti D.R. (2022). Nanoclays in Medicine: A New Frontier of an Ancient Medical Practice. Mater. Adv..

[30] Sandomierski M., Zielińska M., Adamska K., Patalas A., Voelkel A. (2022). Calcium Montmorillonite as a Potential Carrier in the Release of Bisphosphonates. New J. Chem..

[31] Ritto F.P., da Silva E.M., Borges A.L.S., Borges M.A.P., Sampaio-Filho H.R. (2022). Fabrication and Characterization of Low-Shrinkage Dental Composites Containing Montmorillonite Nanoclay. Odontology.

[32] Sandomierski M., Buchwald Z., Voelkel A. (2020). Calcium Montmorillonite and Montmorillonite with Hydroxyapatite Layer as Fillers in Dental Composites with Remineralizing Potential. Appl. Clay Sci..

[33] Wang R., Li H., Guangxu G., Dai N., Jinsong R., Ran H., Zhang Y. (2021). Montmorillonite-Based Two-Dimensional Nanocomposites: Preparation and Applications. Molecules.

[34] De Menezes L.R., Da Silva E.O. (2016). The Use of Montmorillonite Clays as Reinforcing Fillers for Dental Adhesives. Mater. Res..

[35] Dowling A.H., Stamboulis A., Fleming G.J.P. (2006). The Influence of Montmorillonite Clay Reinforcement on the Performance of a Glass Ionomer Restorative. J. Dent..

[36] Giannakas A., Tsagkalias I., Achilias D.S., Ladavos A. (2017). A Novel Method for the Preparation of Inorganic and Organo-Modified Montmorillonite Essential Oil Hybrids. Appl. Clay Sci..

[37] Xue Z., Ma J., Hao W., Bai X., Kang Y., Liu J., Li R. (2012). Synthesis and Characterization of Ordered Mesoporous Zeolite LTA with High Ion Exchange Ability. J. Mater. Chem..

[38] Eliades G., Palaghias G. (1993). In Vitro Characterization of Visible Light-Cured Glass Ionomer Liners. Dent. Mater..

[39] Kakaboura A., Eliades G., Palaghias G. (1996). An FTIR Study on the Setting Mechanism of Resin-Modified Glass Ionomer Restoratives. Dent. Mater..

[40] Nicholson J.W., Brookman P.J., Lacy O.M., Wilson A.D. (1988). Fourier Transform Infrared Spectroscopic Study of the Role of Tartaric Acid in Glass-Ionomer Dental Cements. J. Dent. Res..

[41] (2017). Dentistry—Water-Based Cements—Part 1: Powder/Liquid Acid-Base Cements.

[42] Kantovitz K.R., Carlos N.R., Silva I.A.P.S., Braido C., Costa B.C., Kitagawa I.L., Nociti F.H., Basting R.T., de Figueiredo F.K.P., Lisboa-Filho P.N. (2023). TiO_2_ Nanotube-Based Nanotechnology Applied to High-Viscosity Conventional Glass-Ionomer Cement: Ultrastructural Analyses and Physicochemical Characterization. Odontology.

[43] Gjorgievska E., Nicholson J.W., Gabrić D., Guclu Z.A., Miletić I., Coleman N.J. (2020). Assessment of the Impact of the Addition of Nanoparticles on the Properties of Glass-Ionomer Cements. Materials.

[44] Duarte A.C.A., Pereira R.D.F.C., de Carvalho S.M., da Silva A.G., de Araújo C.T.P., Galo R., Dumont V.C. (2022). Enhancing Glass Ionomer Cement Features by Using the Calcium Phosphate Nanocomposite. Braz. Dent. J..

[45] Ivanišević A., Rajić V.B., Pilipović A., Par M., Ivanković H., Baraba A. (2021). Compressive Strength of Conventional Glass Ionomer Cement Modified with TiO_2_ Nano-Powder and Marine-Derived HAp Micro-Powder. Materials.

[46] Oliveira G.L., Carvalho C.N., Carvalho E.M., Bauer J., Leal A.M.A. (2019). The Influence of Mixing Methods on the Compressive Strength and Fluoride Release of Conventional and Resin-Modified Glass Ionomer Cements. Int. J. Dent..

[47] Fareed M.A., Stamboulis A. (2014). Nanoclay Addition to a Conventional Glass Ionomer Cements: Influence on Physical Properties. Eur. J. Dent..

[48] Nicholson J.W., Coleman N.J., Sidhu S.K. (2021). Kinetics of Ion Release from a Conventional Glass-Ionomer Cement. J. Mater. Sci. Mater. Med..

[49] Awosanya K., Nicholson J.W. (2014). A Study of Phosphate Ion Release from Glass-Ionomer Dental Cements. Ceram.-Silik..

[50] Sayyedan F.S., Fathi M., Edris H., Doostmohammadi A., Mortazavi V., Shirani F. (2013). Fluoride Release and Bioactivity Evaluation of Glass Ionomer: Forsterite Nanocomposite. Dent. Res. J..

[51] Gandolfi M.G., Zamparini F., Degli Esposti M., Chiellini F., Fava F., Fabbri P., Taddei P., Prati C. (2019). Highly Porous Polycaprolactone Scaffolds Doped with Calcium Silicate and Dicalcium Phosphate Dihydrate Designed for Bone Regeneration. Mater. Sci. Eng. C.

[52] Gandolfi M.G., Zamparini F., Valente S., Parchi G., Pasquinelli G., Taddei P., Prati C. (2021). Green Hydrogels Composed of Sodium Mannuronate/Guluronate, Gelatin and Biointeractive Calcium Silicates/Dicalcium Phosphate Dihydrate Designed for Oral Bone Defects Regeneration. Nanomaterials.

[53] Zadpoor A.A. (2014). Relationship between in Vitro Apatite-Forming Ability Measured Using Simulated Body Fluid and in Vivo Bioactivity of Biomaterials. Mater. Sci. Eng. C.

